# Simultaneous Genome-Wide Inference of Physical, Genetic, Regulatory, and Functional Pathway Components

**DOI:** 10.1371/journal.pcbi.1001009

**Published:** 2010-11-24

**Authors:** Christopher Y. Park, David C. Hess, Curtis Huttenhower, Olga G. Troyanskaya

**Affiliations:** 1 Lewis-Sigler Institute for Integrative Genomics, Princeton University, Princeton, New Jersey, United States of America; 2 Department of Computer Science, Princeton University, Princeton, New Jersey, United States of America; 3 Department of Biology, Santa Clara University, Santa Clara, California, United States of America; 4 Department of Biostatistics, Harvard School of Public Health, Boston, Massachusetts, United States of America; Massachusetts Institute of Technology, United States of America

## Abstract

Biomolecular pathways are built from diverse types of pairwise interactions, ranging from physical protein-protein interactions and modifications to indirect regulatory relationships. One goal of systems biology is to bridge three aspects of this complexity: the growing body of high-throughput data assaying these interactions; the specific interactions in which individual genes participate; and the genome-wide patterns of interactions in a system of interest. Here, we describe methodology for simultaneously predicting specific types of biomolecular interactions using high-throughput genomic data. This results in a comprehensive compendium of whole-genome networks for yeast, derived from ∼3,500 experimental conditions and describing 30 interaction types, which range from general (e.g. physical or regulatory) to specific (e.g. phosphorylation or transcriptional regulation). We used these networks to investigate molecular pathways in carbon metabolism and cellular transport, proposing a novel connection between glycogen breakdown and glucose utilization supported by recent publications. Additionally, 14 specific predicted interactions in DNA topological change and protein biosynthesis were experimentally validated. We analyzed the systems-level network features within all interactomes, verifying the presence of small-world properties and enrichment for recurring network motifs. This compendium of physical, synthetic, regulatory, and functional interaction networks has been made publicly available through an interactive web interface for investigators to utilize in future research at http://function.princeton.edu/bioweaver/.

## Author Summary

To maintain the complexity of living biological systems, many proteins must interact in a coordinated manner to integrate their unique functions into a cooperative system. Pathways are typically constructed to capture modular subsets of this dynamic network, each made up of a collection of biomolecular interactions of diverse types that together carry out a specific cellular function. Deciphering these pathways at a global level is a crucial step for unraveling systems biology, aiding at every level from basic biological understanding to translational biomarker and drug target discovery. The combination of high-throughput genomic data with advanced computational methods has enabled us to infer the first genome-wide compendium of bimolecular pathway networks, comprising 30 distinct bimolecular interaction types. We demonstrate that this interaction network compendium, derived from ∼3,500 experimental conditions, can be used to direct a range of biomedical hypothesis generation and testing. We show that our results can be used to predict novel protein interactions and new pathway components, and also that they enable system-level analysis to investigate the network characteristics of cell-wide regulatory circuits. The resulting compendium of biological networks is made publicly available through an interactive web interface to enable future research in other biological systems of interest.

## Introduction

The complexity of cellular activity is driven not only by interactions among genes and gene products, but also by the timing and dynamics of these interactions, the conditions under which they occur, and the many forms that they can take. Proteins interact in many different functional manners with multiple partners - physically in complexes[1] and through modifications[2], [3], synthetically when employed in parallel pathways[4], and in regulatory roles as activators or repressors[5] - and these interaction types combine to form complete molecular pathways. Functional assays such as gene expression, localization, and binding each capture individual aspects of this molecular activity at a global level, but translating the vast amount of resulting genomic data into specific hypotheses at the molecular pathway level has proven challenging. The heterogeneity of gene interactions within each pathway has compounded this difficulty by preventing any one assay from providing a complete biological picture. It is thus critical to integrate large genomic data collections to describe not only the membership of gene products within pathways, but also their construction from the building blocks of individual types of biomolecular interactions.

In this work, we provide the means for investigators to study complete molecular pathways at a whole-genome level as generated from integrated functional genomic data. First, we relate 30 general and specific biomolecular interaction types, such as transcriptional regulation, ubiquitination (and other post-translational modifications), or protein complex formation, in an ontology of interaction types. This ontology is hierarchical, in that a phosphate transfer is perforce a covalent post-translational modification, which is in turn by definition a transient physical interaction, and so forth. Next, we combine this ontology with Bayesian hierarchical classification methodology [6], enabling the simultaneous prediction of genome-wide interaction networks of all of these 30 types from integrated heterogeneous experimental data. Finally, we apply this method to a compendium of ∼3,500 *Saccharomyces cerevisiae* experimental conditions, experimentally validating several of the resulting predictions in glucose utilization, DNA topological maintenance, and protein biosynthesis as described below. This methodology ensures that investigators can take advantage of all available data to accurately identify the entire range of functional interaction types within specific pathways and across an organism's genome.

It is important to contrast this genome-wide system for predicting diverse biomolecular interaction types with previous work predicting specific individual interaction networks. A variety of methodologies have been proposed for inferring regulatory networks [7]–[10], physical interaction networks [11], [12], synthetic interaction networks [13], [14], and other interaction types [15], generally from their respective primary data types (ChIP-chip and -seq, proteomics, double knockouts/knockdowns, etc.) Likewise, other methods have been proposed for heterogeneous genomic data integration [16]–[24], but these almost uniformly focus on either general functional interactions or on specific bimolecular interaction types. This work combines the strengths of these two bioinformatic areas, providing a simultaneous platform with which all data available for a system can be integrated and focused onto specific interaction types, genome-wide and for individual gene products.

We first applied our yeast network compendium to explore two cellular processes, carbon metabolism and cellular transport. This generated many promising interactions involving Snf1, Cmk2, Glc7, Adr1 and Gph1 supported by recent published work. We also suggest several novel pathway connections, such as the interplay between the glycogen breakdown and glucose utilization pathways, by systematically layering multiple different interaction types. To experimentally validate a collection of our predicted yeast interactions, we focused on the synthetic lethal interactions, where double knockouts result in lethality, predicted among proteins involved in DNA topological change and regulation of protein biosynthesis. Highly ranked 20 protein pairs, 10 pairs from each pathway, were hypothesized to be synthetically lethal, and we experimentally confirmed 14 of these pairs (70%). Furthermore, we evaluated our posttranslational modification predictions based on recent experimental results on 173 protein pairs, resulting in a prediction AUC over 0.8. In an analysis of the systems-level global and local network structures of our interactomes, we observed differential usage of several recurring subgraphs, providing insight into the functional design principles of pathway components. Finally, we provide a web-based interface to explore all 30 yeast interaction networks at http://function.princeton.edu/bioweaver. This will allow investigators to interactively survey and generate hypotheses from the diverse interaction types comprising the *S. cerevisiae* cellular circuitry.

## Results

**Figure 1:**
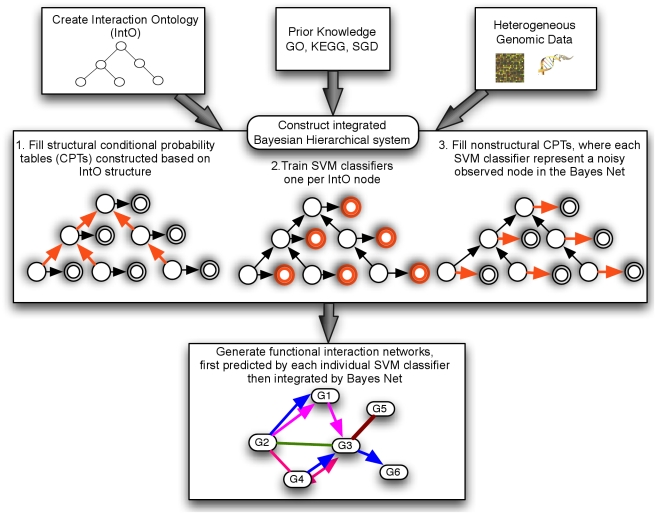
Overview of our integrated Bayesian hierarchical system for inferring diverse interaction networks. An interaction ontology was constructed categorizing gene interaction types. A corresponding Bayesian network was constructed in which each node represents one interaction type. This network's structural parameters, P[*parent node label* |*child node labels*], were first determined using prior knowledge from GO [36], KEGG [59], SGD [56], and other curated sources. Second, individual SVM classifiers were trained to predict each interaction type in isolation using heterogeneous data sources. Third, the non-structural Bayesian network parameters, P[*true latent node label* |*SVM output*], were filled by relating each observed SVM classifier to a latent interaction type membership node using cross validation. Finally, to generate new predictions, a gene pair's interaction type is first predicted by the SVM classifiers and then hierarchically resolved by finding the most probabilistically consistent set of label assignments corresponding to the latent nodes in our Bayesian network.

We present a general methodology for integrating large, diverse genomic data compendia to simultaneously predict multiple biomolecular interaction network types (physical, genetic, regulatory, etc.; Figure 1). We applied this methodology to ∼3,500 *S. cerevisiae* experimental conditions to generate 30 whole-genome networks describing predicted gene and gene product interactions in yeast. We first evaluated these predictions quantitatively using cross-validation, achieving AUCs over 0.7 for most interaction types. More qualitatively, we examined a set of predicted molecular linkages of diverse types between glycogen breakdown and glucose utilization genes, which were validated by recent literature. Finally, we experimentally confirmed 14 of 20 predicted novel synthetic lethal interactions in the DNA topological change pathway.

### Evaluating the accuracy of predicted S. cerevisiae biological networks

**Figure 2:**
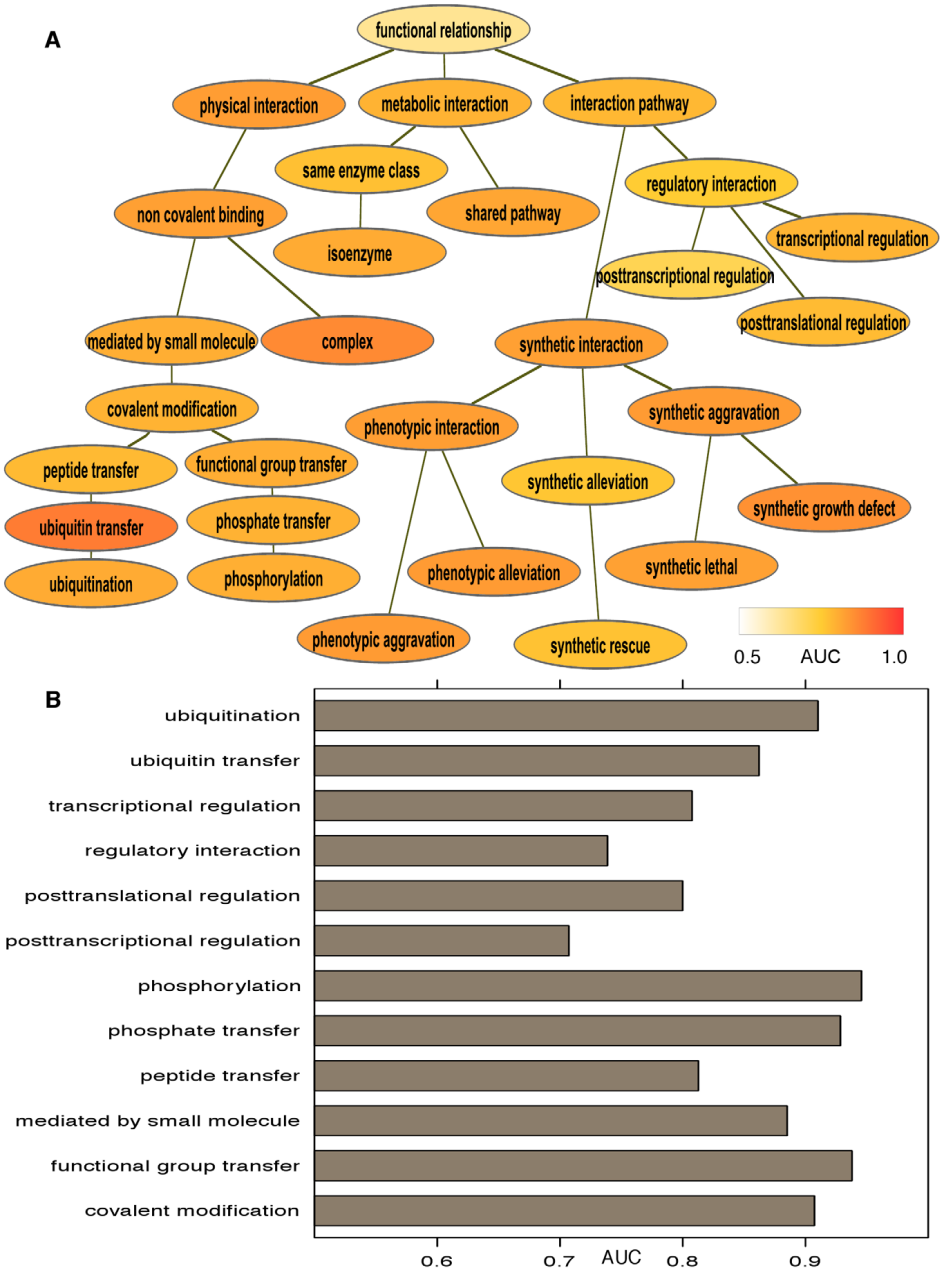
Performance evaluation of inferred networks. We predicted 30 *S. cerevisiae* interaction networks, each representing one interaction type. A) To evaluate the overall accuracy of these networks, we withheld ∼30% of the genes in our gold standard as a test set. Performance on this test set averaged an AUC of 0.79 across all interaction types in the ontology; see Text S1 for individual ROCs. B) To specifically assess the accuracy with which interaction directionality was predicted (as opposed to the presence/absence of interactions in part A), we tested the frequency with which an interaction's correct direction was ranked above its incorrect direction in each of the 12 directed interaction networks. These results are uniformly well above random (0.5), supporting our ability to accurately predict both the presence and the directionality of many specific types of protein interactions.

We predicted a compendium of biomolecular interaction networks by integrating diverse yeast genomic data using a multi-label hierarchical classification system ([6], Figure 2A). As briefly outlined in Figure 1, we first independently predict each interaction type using specifically trained SVM classifiers. Next, it is desirable to avoid making inconsistent interactome predictions due to noisy data, e.g. predicting that two genes share a regulatory relationship without occurring within the same pathway. In order to share information among classifiers for related interaction types in a principled manner, each SVM's predictions are treated as noisy observations. The final set of labels for each gene pair is then derived by finding the maximum likelihood assignment of interaction labels by integrating these observations in a Bayesian graphical model.

Based on ∼30% heldout test data, our average AUC over all 30 interaction types was 0.79, with minimal variations in performance across the interaction ontology (Figure 2A, Figure 1 in Text S1). The most general interaction type, *functional relationship*, also incurred the lowest AUC of 0.63, which remains comparable to state-of-the-art functional interaction prediction systems [25]. In order to quantify the contribution of our hierarchical Bayesian system relative to predicting disparate biomolecular interaction types in isolation, we compared the accuracy of each individual SVM classifier to that of the complete system. For all 30 predicted interactomes, the Bayesian hierarchy showed increased AUC scores, averaging +0.076 and ranging from a minimum of +0.011 to a maximum of +0.166. For example, posttranslational regulation improved from 0.61 to 0.77, while phosphorylation increased from 0.67 to 0.79. (full ROC curves for all interaction networks can be found in Text S1). In combination, these two evaluations suggest that this methodology can accurately leverage large genomic data collections to simultaneously infer a diversity of genome-wide interaction networks.

### Accurate prediction of directed interaction networks

Many gene interactions are directional and thus asymmetric, e.g. phosphorylation or ubiquitination, in which the two interactors take on distinct source and target roles. It is thus important to correctly infer not only the presence or absence of these directed interactions, but also the correct directionality. Specifically, for each directed interaction type, we constructed a list of all edges ranked by predicted probability; we then compared the rank of the correct interaction direction relative to the incorrectly flipped interaction between the same two genes (Figure 2 in Text S1). Using this as a true- and false-positive rate criterion, we were able to predict the correct direction of gene interactions with average AUC of 0.85 over the 12 directed networks (maximum 0.94, minimum 0.70). This indicates that this methodology can accurately recover not only overall pathway structure, but also the upstream and downstream effects of individual gene products within molecular pathways.

### Predicted interactions provide mechanistic insight into the yeast glycolysis pathway

Simultaneous inference of biomolecular networks for many different interaction types allows the generation of very specific novel hypotheses. As a first example, we detail a combination of transcriptional, genetic, post-translational, and metabolic interactions among gene products coordinating glycogen breakdown and glucose utilization in yeast.

**Figure 3:**
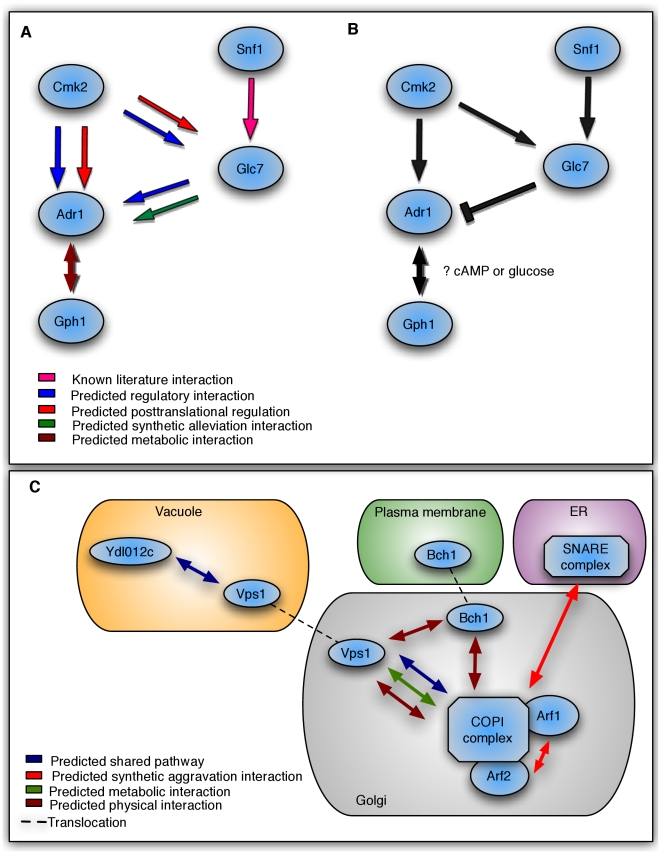
Examining the mechanisms of protein interactions within the yeast carbon metabolism and cellular transport pathways. A) Predicted interactions of four specific types combined to assemble B) (arrows in black representing our final predicted pathway interactions) a pathway connecting the transcription factor Adr1 involved in carbon metabolism process to its regulatory input Snf1. This generates two concrete hypotheses suggesting, first, cross-talk between the calmodulin- and Snf1-dependent pathways via Cmk2 phosphorylating Glc7. Second, we also predict coordinated regulation between the glycogen breakdown and glucose utilization pathways through a metabolic interaction between Adr1 and Gph1. C) Previously known and newly predicted interactions in yeast protein transport connecting the plasma membrane, vacuole, golgi and ER. We propose a regulatory competition between the Arf1 and Vsp1 GTPases for Bch1 functionality that is likely regulated by GTP availability, which itself is known to be regulated by protein sorting events in the cell. These predictions also hypothesize that YDL012c may be involved in regulating Vps1 activity.

As shown in Figure 3, Adr1 is an important transcription factor involved in carbon metabolism in *Saccharomyces cerevisiae*. It has many known regulatory inputs [26], one of which is the glucose-responsive kinase Snf1, and what proteins transmit this regulatory information has been under investigation for some time. By examining different classes of predicted interactions with Adr1 and other proteins *not* in our gold standard (Figure 3A), we first identified regulatory and genetic interactions between the protein phosphatase Glc7 and Adr1. Specifically, our prediction of a synthetic alleviating interaction between Glc7 and *adr1* mutants places it upstream of Adr1 in this pathway. This combination of interactions is almost always associated with an upstream inhibitory regulator, consistent with the known biological role of Glc7 as a protein phosphatase that removes activating phosphorylations [27].

The predicted yeast networks also hypothesized post-translational regulatory interactions between Cmk2 and both Adr1 and Gkc7 (Figure 3A). This three-protein network creates a feed-forward regulatory motif in which Cmk2 simultaneously activates Adr1 as well as its inhibitor Gkc7, creating a time-delayed inactivation of Adr1. These interactions are supported by a recently published paper [26] linking the calmodulin- and Snf1-dependent pathways to Adr1 regulation. Our predicted pathway takes these results a step further by identifying which of the three calmodulin-dependent kinases (Cmk2) is responsible [28]. Finally, a novel metabolic interaction was predicted between Adr1 and Gph1, the only high scoring interaction of its type for Adr1. Like Adr1, Gph1 is involved in glucose metabolism by glycogen breakdown, and both are regulated by the metabolites glucose and cAMP [29]. A metabolic interaction between Adr1 and Gph1, combined with the known regulation of these genes by glucose and cAMP, suggests that coordinated regulation is occurring between the glycogen breakdown and glucose utilization pathways and is transcriptionally controlled by Adr1.

### An inferred pathway incorporating physical, genetic, and metabolic interactions spans cellular compartments in yeast protein transport

Protein sorting and trafficking is an essential function of eukaryotes and requires numerous multi-subunit complexes to ensure the proper localization and secretion of proteins (Figure 3C, [30]). At the early stages of this process, the two major transport pathways from the endoplasmic reticulum (ER) to the Golgi are governed by the SNARE and COPI complexes [30]. We predicted synthetic interactions between these pathways (e.g. synthetic aggravation for Arf1-Sec18 and synthetic alleviation for Sec27-Uso1) that are supported by known genetic interactions[31], [32]; Arf1 and Arf2 are a representative example, as they are considered functionally redundant GTPases, and each COPI complex contains either Arf1 or Arf2 [33].

Later in the pathway, Bch1 is a member of the ChAP family of proteins, which direct cargo bound to COPI complexes in the Golgi to their destinations such as the plasma membrane [34]. We predict a physical interaction between Bch1 and the COPI complex that is well established in the literature but was not part of our gold standard. Likewise, Vps1 serves a similar function for vacuole targeting [35], and our predictions of its physical and shared pathway interactions with COPI are supported by the literature [34].

Novel hypotheses in Figure 3C include the predicted physical interaction between Bch1 and Vps1, suggesting competition between the Sec27-Arf1 and Vps1 complexes for the Bch1 sorting function (also supported by a metabolic interaction between Sec27-Arf1 and Vps1). Both Vps1 and Arf1 are GTPases that must hydrolyze GTP to perform their roles in protein sorting [33]. Thus, this predicted pathway hypothesizes a competition between the Arf1 GTPase and Vsp1 GTPase for Bch1 that is likely regulated by GTP availability. Similarly, the uncharacterized membrane-bound protein YDL012c is placed in the same pathway as Vps1, suggesting that the former may be involved in regulating Vps1 activity. By highlighting just a few of our predicted interactions in the protein sorting pathway, we demonstrate the potential for generating hypotheses used to drive novel biological discoveries.

### Experimental validation of predicted interactomes

**Figure 4:**
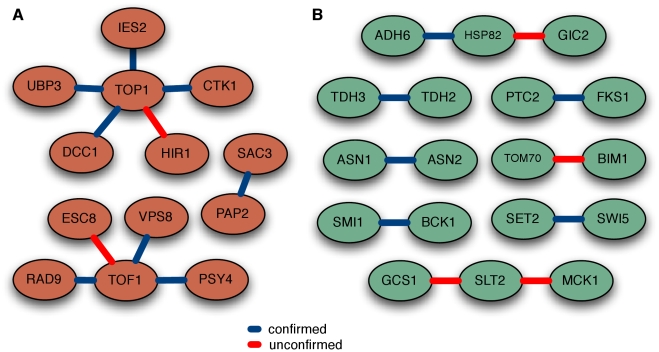
Experimental validation of predicted synthetic lethal interactions. Experimentally tested synthetic lethal hypotheses in the yeast A) DNA topological change and B) regulation of protein biosynthesis processes. A total of 20 gene pairs from our predicted synthetic lethality networks were experimentally tested using the SGA platform [4], [13]. We confirmed 14 of these interactions (70%), 8 in DNA topological change and 6 in protein biosynthesis. Several of the remaining unconfirmed pairs (e.g. GCS1 and SLT2; see main text) show additional evidence of condition-specific synthetic lethality.

To experimentally evaluate the accuracy of a subset of our predicted interactions in a directed manner, we focused on the DNA topological change and protein biosynthesis regulation processes in *S. cerevisiae* [36]. 20 synthetic lethality interactions predicted with high probability were experimentally tested using SGA technology [4], [13], with the results summarized in Figure 4. 14 gene pairs (70%) were validated, 8 involved in DNA topological change and 6 in the regulation of protein biosynthesis. Several of the remaining 6 unconfirmed interactions may be synthetic lethal under different conditions. For example, GCS1 and SLT2 deletions both individually decreased resistance to ethanol stress [37], and similar conditions might elicit synthetic lethality. Based on a total of ∼100,000 pairs estimated to have been synthetically lethal in yeast of a possible ∼18 million (0.05%) [13], our predictions are a clear improvement over the baseline rate for novel discovery.

As an additional evaluation, we collected 24 recent publications containing a total of 173 experimentally confirmed post-translationally regulated protein pairs (see Text S2 for the list of publications). None of these interactions was present in our training standard. Evaluating on this set, our Bayesian hierarchical system achieved an AUC of 0.802, demonstrating its ability to accurately predict novel, experimentally verifiable post-translational regulation interactions on a whole-genome scale. This accuracy is comparable to our initial cross-validation AUC of 0.778, indicating that our evaluation provides a reasonable estimate of the expected experimental verification rate for novel predictions.

### Systems level view of cellular interactomes

**Figure 5:**
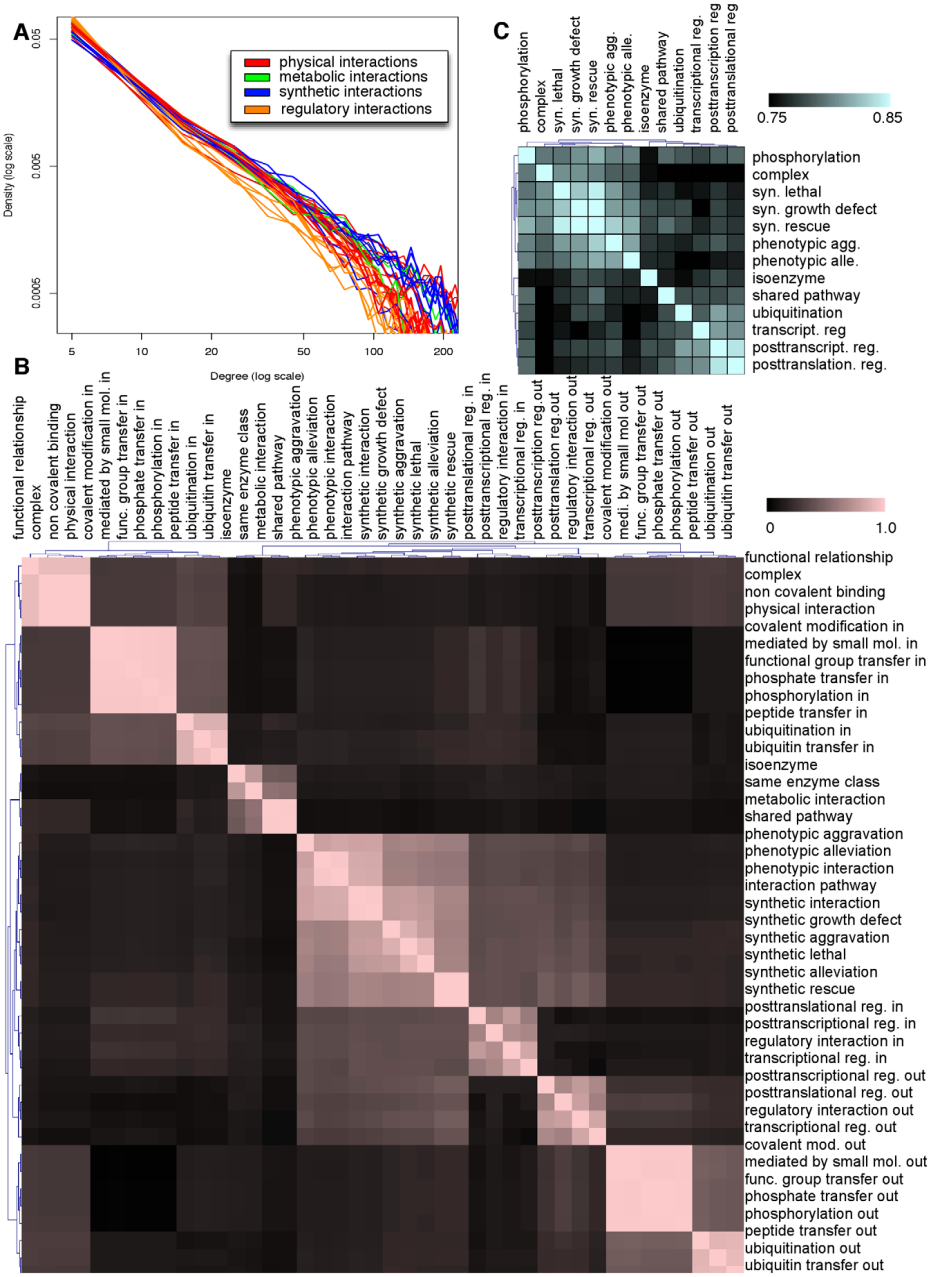
Systems-level analysis of inferred networks. In all cases, continuously weighted networks were binarized by choosing an edge cutoff three standard deviations above mean, retaining ∼1% of all edges. A) The degree distribution for all 30 of our predicted interactomes agrees strongly with a scale-free network topology. B) Conditional probabilities for a gene to appear in the top 5% of each pair of networks' degree distributions. Similarity indicates that a pair of networks share the same high-connectivity genes and thus represent functional activity carried out by similar sets of proteins. C) Graphlet degree distributions compared using the GDD metric between the 13 leaf interactomes in our interaction ontology. Network pairs with greater similarity demonstrate related local network topologies, suggesting that comparable functional modules might be employed in the two interactomes (e.g. between phosphorylation and synthetic interactions or ubiquitination and post-translational regulation).

This rich compendium of inferred interaction types provided an opportunity to analyze systems-level network features genome-wide at multiple levels of biomolecular activity. In particular, we examined the network structural characteristics that potentially help to define the functional roles of each interactome. Biological networks have been proposed to exhibit a scale free topology [38], implying a power-law degree distribution. Previous studies have detected such distributions based on partial networks and single interactomes [39]. Here (Figure 5A), we observe a scale-free degree distribution very robustly in all 30 interaction types. However, the high-degree hubs in each interactome do differ, reflecting the distinct functional activities carried out by each network type. To verify this, we analyzed the extent of the overlap of high-connectivity genes (in the top 5% of the degree distribution) between the networks for each pair of interactomes (Figure 5B; directed interactomes were divided into separate in- and out-degree comparisons). The major clusters show distinct functional similarity, correctly reflecting the similarities captured by our interaction ontology: transient and nontransient physical interactions each group together, synthetic interactions cluster, and so forth. Beyond confirming the structure of the ontology, this also captures relationships such as the sharp divide between regulatory in- and out-degree (the most regulated genes are not themselves high-level regulators with many targets) and a tendency for regulatory hubs to incur more synthetic interactions than expected.

Degree distribution captures a global description of each network, while analysis of small recurring subgraphs has been proposed to describe local network properties [40], [41]. We investigated the enrichment of two types of subgraphs, network motifs and graphlets, in our interactomes. First, network motifs are small directed subgraphs that have been found to recur in a growing number of organisms [42]–[44]. In our 12 directed interaction networks, the feed forward loop motif showed significant enrichment (relative to a random background; see Text S1) consistent with previous studies on the yeast transcription factor network [41]. Feed forward loops are known to accelerate or delay the response of a input signal [45], suggesting in this context a much wider usage of dynamic information processing than has been previously reported in regulatory networks[46]–[48].

A second approach to exploring the local structure of biological networks is to examine graphlet degree distributions [40]. Graphlets are small non-isomorphic subgraphs, and a graphlet's degree for a given node is defined as the number copies of that graphlet to which it is incident. For example, the number of triangle motifs touching a particular node represents its 3-node graphlet degree. Compared to network motifs, for which enrichment can be difficult to detect due to the complexity of the baseline null distribution[49], graphlet analysis may have a higher sensitivity towards infrequent subgraphs. Thus, as a complementary analysis, we computed the graphlet degree distributions for all two to five node graphlets for the 13 specific leaf node interactomes in our interaction ontology (Figure 5C). We compared the graphlet degree distributions between these interactomes, demonstrating a clear divergence in the local network structure between subclasses of metabolic, regulatory and synthetic interactions. Unlike the comparison of high-degree genes, this also captures unexpected similarities between disparate interaction types: phosphorylation and ubiquitination, for example, are siblings in the interaction ontology and represent comparable mechanisms of post-translational modification. The former's local network topology is more similar to that of synthetic interactions, however, while ubiquitination is more strongly regulatory. This differentiating pattern between ubiquitination and phosphorylation provides a base for intriguing network hypotheses for further investigation. One potential explanation could be due to the differing mechanistic activities where ubiquitination is most often employed exclusively as a regulatory mechanism to degrade active proteins, whereas phosphorylation serves both regulatory and dynamic information processing roles [50].

## Discussion

The increasing abundance of genomic data has opened up countless new possibilities for systems-level biological perspectives, but its increasing complexity impedes the understanding of specific cellular circuitry at a mechanistic level. Here, we provide a method with which very large experimental data compendia can be integrated to predict 30 specific biomolecular interaction types at a genome-wide scale. By applying this to more than ∼3,500 experimental conditions in yeast, we have evaluated these predictions at an average AUC of 0.79, validated 70% of experimentally tested synthetic lethal interactions, and proposed novel transcriptional, genetic, post-translational, and metabolic interactions in the yeast carbon metabolism and cellular transport pathways.

As described above, the investigation of specific *S. cerevisiae* biology in the processes of glucose utilization and protein trafficking demonstrates the use of these interactomes to reconstruct complete pathways. In many instances, experimental biologists are faced with the task of designing experiments to target a specific set of genes. By simultaneously hypothesizing all types of biomolecular interactions in which a group of gene products may be involved, this methodology can be used to select both the proteins to be assayed and the assays that may be most informative. Prior approaches inferring these interaction types in isolation mask this information and may even be inconsistent; how might a biologist interpret predictions that two proteins physically interact, but that they are not part of the same pathway? Such inconsistencies are avoided by simultaneous ontology-based inference, allowing underlying experimental data to be integrated into a consistent description of a cellular system.

To our knowledge, there has been no other method that simultaneously enables researchers to leverage high-throughput data in an interaction-type-specific manner within an ensemble setting. Successful focused attempts to predict specific interaction types have shown comparable AUCs to our results [51], [52], which could be incorporated into a framework like this as base classifiers during future work (instead of the SVMs utilized in this study). Recent “functional coupling” predictions [20] are also related, but fall short of pathway-level interaction predictions, mainly due to a lack of the crucial directional information needed to infer bimolecular pathways. These frameworks typically also do not resolve inconsistencies among predicted interaction type labels that can hinder pathway reconstruction and experimental follow up.

Ultimately, compendia of inferred interaction networks can be used to explicitly construct and understand distinct cellular pathways. By investigating and confirming different interaction types suggested by our system, investigators can stitch together both new pathways and new interconnections between existing ones. This process can be applied in any organism for which diverse genome-scale data is available - a situation that is only becoming more common. We believe that our work can leverage this diversity of experimental results that might otherwise be underutilized, helping to spur new functional discoveries in organisms beyond yeast. Finally, all of our predicted networks are made publicly available through an interactive tool at http://function.princeton.edu/bioweaver for investigators to explore their own biological areas of interest.

## Materials and Methods

We developed an integrated method for concurrently predicting multiple protein interaction types. This method integrates large and diverse collections of functional genomic data in the context of a biomolecular interaction ontology. Each gene interaction type in the ontology is first predicted using an SVM classifier by integrating ∼3,500 experimental conditions from expression, colocalization, regulatory, and other yeast experimental data (withholding data types directly related to the interaction type being predicted; see below). These isolated interactomes are then reconciled using a hierarchical Bayesian framework to obtain the most probable set of consistent labels for each gene pair within the hierarchy of our interaction ontology. Using this system, we generated 30 *S. cerevisiae* interactomes, with which we validated several mechanistic interaction predictions in carbon metabolism, cellular transport, and 14 new synthetic lethal interactions in DNA topological change and protein biosynthesis.

### Interaction ontology construction

We constructed an interaction ontology focused on categorizing gene pair relationships. This is similar in spirit to the Gene Ontology (GO) [36], which curates individual proteins' molecular functions, biological roles, and subcellular localizations. Our interaction ontology contains a total of 124 terms and integrates information from existing interaction catalogs [53], [54], the EBI [55], and SGD [56]. The ontology's three major branches are metabolic, interaction pathway, and physical interactions. Metabolic interactions describe protein pairs linked in metabolic pathways, such as isoenzymes or enzymes that catalyze adjacent reactions. Physical interactions include covalent or non-covalent binding, e.g. stable complexes or transient post-translational modifications. Pathway interactions include more conceptual relationships between genes in a pathway, such as regulation or synthetic interactions. We selected the 30 nodes in our interaction ontology with more than 70 annotations (as described below) to include in this evaluation, and the complete ontology with descriptions of each term is provided in Text S1 and Text S3.

### Gold standard construction

There exists no comprehensive curated gold standard repository for all types of gene pair interactions. For the 30 interactomes evaluated here, we assembled a gold standard for each type from various sources. SGD interaction labels were used for all terms under the physical and pathway interaction terms [56]. Additional transcriptional regulation annotations were obtained from the high confidence set from [57]. Co-complex annotations were obtained from gene pairs in the GO Slim term *PROTEIN_COMPLEX* [58]. Pairs included in terms under metabolic interaction were obtained from reactions in the KEGG database [59]. For the topmost node, functional relationships, we used positive examples from the biological process branch of GO [60]. When possible, we further manually curated gene pairs to more specific terms based on literature examination. Manual curation was performed to annotate ubiquitination interactions based on SGD curated interaction publications and also to cross annotate experimentally validated covalent modification branch examples to regulatory interaction branch terms. The directionality of the gold standards was derived directly from the inherent high throughput experiments (e.g. kinases to targets). All gene pairs annotated to a term were propagated such that they were included as positive interactions for all ancestor terms. This resulted in a total of 1,333,014 unique positive labels across 30 terms (individual terms are detailed in Text S1).

This process established positive interactions for each term in our interaction ontology. For supervised machine learning (such as our SVM-based method described below), negative examples are also required. As protein interactions are sparse, we randomly selected a number of negative gene pairs for each term's gold standard equal to the number of positive interactions [61]. Additionally, to assess the accuracy of our directed interaction predictions, we used negative gene pairs identical to the positive examples but with inverted directionality. Finally, for evaluating predictions on new post-translational regulation completely unrelated to our training gold standard, we selected 173 additional gene pairs from 24 recent publications (see Text S1).

Evaluation was performed by randomly excluding ∼%30 of the genes for each interaction type during training. That leads to a group of genes that are not in the training set and established a test set of interactions containing at least one gene from this exclusive gene set. The remaining pairs were used for SVM training and for parameter estimation in the Bayesian network. We used area under the receiver operator characteristic (ROC) curve (AUC) for evaluation as detailed in Text S1.

### Data sources and preprocessing

As training data for each interaction type, we used subsets of a data compendium consisting in total of microarray, colocalization, protein domains, transcription factor binding sites, and sequence similarity. For each interaction type to be predicted, experimental data closely related to the output was excluded (e.g. TF binding sites for regulatory relationships). 78 yeast microarray datasets were included, comprising 3,516 conditions (see Text S2). Missing values in these datasets were imputed using KNNImpute [62] with *k* = 10, and genes with more than 30% missing values were removed.

For machine learning, one feature was constructed per expression condition as follows. For directional gene pair interaction types such as phosphorylation, we evaluated various methods and found *x_i_*-*x_j_* to provide optimal performance, where *x_i_* and *x_j_* are the expression values of gene *i* and *j* in condition *x*. When predicting non-directional interaction types such as physical interaction, we used |*x_i_*-*x_j_*|, the absolute value of the subtracted expression values.

Colocalization data for 22 different cell compartments [63] and automatically determined protein family information from Pfam B [64] were both included as binary features (true if both genes in a pairs shared localization or a protein family). TRANSFAC data [65] was incorporated using the Euclidian distance between the two gene's binding site profiles across 211 transcription factors. Sequence similarity between the two genes in each pair's 1,000 bp upstream and 1,000 bp downstream was scored as the sequence alignment E-values from all-against-all BLAST outputs.

### Algorithm

We developed an integrated method for predicting diverse protein interactions, based on a multi-label hierarchical classification formulation we have previously applied to gene function prediction in both yeast and mouse [6], [66]. First, for each interaction type, we trained 10 separate SVM classifiers. We use bagging (bootstrap aggregation, [67]) to combine these and improve generalization, training each individual SVM classifier on a bootstrapped subsample of its interaction type's complete gold standard. We thus begin with a total of 300 SVM classifiers for our 30 interaction types in yeast, and each interaction type's group of 10 SVM outputs were averaged (bagged) to produce a non-hierarchically-resolved predicted interactome.

Next, a Bayesian network was constructed based on the structure of the interaction ontology. First, we modeled each interaction type's bagged SVM output *i* as a random event *Y_i_* taking discrete values binned by five standard deviations above or five below the training set mean. Each SVM's predictions in isolation were treated as a noisy observation of a latent event *X_i_* representing the true, binary interactions and non-interactions of each type *i*. Each *Y_i_* was considered to be dependent only on its corresponding *X_i_*, and each *X_i_* was dependent only on its set of children {*X_j_*, ..., *X_k_*} in the interaction ontology, resulting in the “decorated tree” Bayesian network structure seen in Figure 1 and in [6]. Given this structure, conditional probability table parameters for P(*Y_i_*|*X_i_*) were learned using maximum likelihood from interaction type *i*'s training data. Finally, parameters for P(*X_i_*|*X_j_*, ..., *X_k_*) were fixed to constrain the hierarchical semantics of the ontology. If a pair is annotated to any child in {*X_j_*, ..., *X_k_*}, it must also be of interaction type *i*, making P(*X_i_* = 1|*X_j_* = 1)  =  ...  = P(*X_i_* = 1|*X_k_* = 1)  = 1. The remaining parameters P(*X_i_* = 1|*X_j_* = 0, ..., *X_k_* = 0) were inferred using maximum likelihood by counting the corresponding training labels. Finally, Laplace smoothing was used to improve parameter robustness.

### System level network analysis

All 30 interactomes were converted into binary interaction networks by setting a threshold of 5 standard deviations above the mean edge probability, retaining ∼1% of all edges. The degree of each gene was counted in this binarized network. The overlap between each pair of interactomes' high-connectivity genes was computed as the probability of a gene *g* being in the top 5% of interactome *N*_1_'s degree distribution(*Q*_i_(*N*_j_), defined as genes in the top *i* percent degree distribution of interactome *N*_j_) given that it was in *N*_2_'s: P[*g* in *Q*_0.05_(*N*_1_)|*g* in *Q*_0.05_(*N*_2_)]. For each of the 30 interactomes *N*_2_, we generated a sorted gene list by edge degree; for directed interactomes, separate lists were generated for in- and out-degree. Next, we counted the number of shared genes in the top 5% of edge degree in the target interactome *N*_1_. Finally, hierarchical clustering was used to generate clusters of shared high degree genes.

Network motif enrichment analysis was carried out using FANMOD [68]. Searches were conducted for 3-node motifs using a sampling method with probability parameters of 0.6, 0.5, 0.4 and compared to 500 random networks generated using an edge swapping process preserving each gene's degree. Computational complexity precluded analysis of 4-node motifs. Graphlet degree distributions were calculated using GraphCrunch [69]. For each interactome, 73 graphlet degree distributions were generated, each representing a unique distribution of 2-5 node graphlets. Comparison between graphlet distributions was performed using the *GDD agreement* metric, defined as the average normalized distance to provide robust comparisons [40], [69].

### Implementation

All software was implemented using the Sleipnir library [70], which interfaces with the SVM^perf^ software [71] for linear kernel SVM classifiers (the error parameter C was set to 20 for these experiments). Bayesian network inference used the Lauritzen algorithm [72] as implemented in the University of Pittsburgh SMILE library [73].

### Experimental validation of synthetic lethal pairs

20 gene pairs predicted to synthetically interact [56] with high probability were selected from the DNA topological change and regulation of protein biosynthesis pathways in yeast (as defined by GO [36]). Synthetic Genetic Array (SGA) technology [4], [13] was applied to these pairs by combining either non-essential gene deletion mutants or conditional alleles of essential genes in haploid yeast double mutants. The query mutant strain for each pair of genes (harboring SGA-specific reporters and markers) was crossed to the complementary single mutant strain. Mating to the non-essential gene deletion collection was followed by meiotic recombination and selection of haploid meiotic progeny, resulting in an output array of double mutants grown in rich medium. Fitness was assessed by comparing this double mutant colony size to the sizes of single mutant colonies, which were assessed for significance as described in [4], [13]. A p-value threshold of 0.05 was used to determine the final confirmed synthetic lethal pairs (the full table of p-values can found in Text S1).

## Supporting Information

Text S1 Additional description of the results and methods from the paper.
(1.12 MB DOC)

Text S2 The microarray dataset list used in our functional integration.
(0.00 MB TXT)

Text S3 Interaction ontology files - includes OWL ontology format file and visual ontology PDF file
(0.03 MB ZIP)
