# Supplementary material for: Simultaneous Genome-Wide Inference of Physical, Genetic, Regulatory, and Functional Pathway Components
Source: PLoS Comput Biol. 2010 Nov 24;6(11):e1001009. doi: 10.1371/journal.pcbi.1001009 (PMC2991250; doi:10.1371/journal.pcbi.1001009)
Supplement: Text S1 — Additional description of the results and methods from the paper. (1.12 MB DOC) [file pcbi.1001009.s001.doc]

**Text S1**

***Supplementary Information***

Table of contents

Table 1. Interaction ontology term descriptions. ……….……………………………………….…..……1
Figure 1. AUC comparison after and before Bayesian modification.……………………………….……3
Table 2. Experimental synthetic lethal pairs with p-values.………………………………………………4
Table 3. Gold standard example count.………………………………………..………………………….5
Table 4. Experimental posttranslational regulation publication list……………….…………..………….6
Figure 2-31. ROC curves for 30 interaction terms………...………………..………………………...7~36
Table 5. Feed forward loop motif z-scores………………………………………………………………37
Figure 32. The distribution of # protein pairs per # interaction leaf term labels. ……………………….38
List of microarray data sets.………………………………………..………......................................Text S2
Interaction ontology……………………………………………………………………………….... Text S3

For all 30 interaction terms ROC curves and description of the terms, Interaction ontology file and list of microarray data sets used can also be found at <http://function.princeton.edu/bioweaver/supplement.html>.

| **Interaction ontology term** | **Description** |
| --- | --- |
| Complex | Any macromolecular complex composed of two or more polypeptide subunits. |
| covalent_modification | Proteins regulated by transfer or remove of a molecule or atom from a donor to an amino acid side chain that serves as the acceptor of the transferred molecule or regulating an enzyme by altering the amino acid sequence itself by proteolytic cleavage. |
| functional_group_transfer | Transfer or removal of a functional group. |
| functional_relationship | Two proteins function in same biological process. |
| interaction_pathway | Two genes interact within a pathway level, including post-transcriptional, transcriptional and post-translational regulation or the functional dependency such as synthetic interactions. |
| isoenzyme | Enzymes that differ in amino acid sequence but catalyze the same chemical reaction. |
| mediated_by_small_molecule | Two proteins where a small molecule is involved as part of a protein modification. |
| metabolic_interaction | Functionally associated at the metabolic level. |
| non_covalent_binding | Two proteins interact in a non covalent nature. |
| peptide_transfer | Transfer peptide to protein. |
| phenotypic_aggravation | Mutation or over expression of one gene results in suppression of any phenotype (other than lethality/growth defect) associated with mutation or over expression of another gene. |
| phenotypic_alleviation | Mutation or over expression of one gene results in enhancement of any phenotype (other than lethality/growth defect) associated with mutation or over expression of another gene. |
| phenotypic_interaction | Mutation or over expression of one gene results in alteration of any phenotype (other than lethality/growth defect) associated with mutation or over expression of another gene. |
| phosphate_transfer | Addition/removal of a phosphate (PO4) group to/off a protein. |
| phosphorylation | Addition of a phosphate (PO4) group to a protein. |
| physical_interaction | Two proteins physically interact. |
| posttranscriptional_regulation | Post-transcriptional regulation is the control of gene expression at the RNA level. |
| posttranslational_regulation | Post-translational regulation refers to the control of the levels of active proteins. |
| regulatory_interaction | A gene regulates a gene either at the RNA, protein or transcription level. |
| same_enzyme_class | Two enzymes that share the same enzyme class. |
| shared_pathway | Two proteins are closely involved in a pathway |
| synthetic_aggravation | Mutation or deletion of one gene aggravates the effect of a strain mutated/deleted for another gene. |
| synthetic_alleviation | Mutation or deletion of one gene alleviates the effect of a strain mutated/deleted for another gene. |
| synthetic_growth_defect | Interaction is inferred when mutations in separate genes, each of which alone causes a minimal phenotype, result in a significant growth defect under a given condition when combined in the same cell. |
| synthetic_interaction | Interaction in which a combination of mutations in two or more genes of a single strain results in a phenotype that is different in degree or nature from the phenotypes conferred by the individual mutations. |
| synthetic_lethal | Mutations or deletions in separate genes, each of which alone causes a minimal phenotype, result in lethality when combined in the same cell under a given condition. |
| synthetic_rescue | Mutation or deletion of one gene rescues the lethality or growth defect of a strain mutated/deleted for another gene. |
| transcriptional_regulation | Transcriptional regulation is the change in gene expression levels by altering transcription rates. |
| ubiquitination | The post-translational modification of a protein by the covalent attachment of one or more ubiquitin monomers. |
| ubiquitin_transfer | The post-translational modification of a protein by the covalent attachment or removal of one or more ubiquitin monomers. |

Table 1. Interaction ontology term descriptions.

Figure 1. AUC comparison after and before Bayesian modification. Each point represents an interaction ontology term where points above the diagonal line represent accuracy improvement by our integrated Bayesian system over the SVM classifier output.

| DNA topological change | |  |
| --- | --- | --- |
| YAL002W | YNL273W | 9.44E-07 |
| YNL273W | YBL046W | 3.87E-02 |
| YOL006C | YKL139W | 2.61E-02 |
| YNL273W | YOL017W | 9.48E-02 |
| YDR159W | YOL115W | 1.39E-04 |
| YCL016C | YOL006C | 2.30E-02 |
| YNL273W | YDR217C | 1.60E-31 |
| YER151C | YOL006C | 1.60E-02 |
| YBL008W | YOL006C | 3.00E-01 |
| YOL006C | YNL215W | 5.03E-04 |
| Regulation of protein biosynthesis | | |
| YMR318C | YPL240C | 1.65E-02 |
| YGR229C | YJL095W | 1.99E-10 |
| YGR192C | YJR009C | 1.75E-132 |
| YDL226C | YHR030C | 3.73E-01 |
| YPR145W | YGR124W | 1.72E-02 |
| YPL240C | YDR309C | 3.37E-01 |
| YHR030C | YNL307C | 1.64E-01 |
| YER089C | YLR342W | 3.59E-02 |
| YNL121C | YER016W | 1.95E-01 |
| YJL168C | YDR146C | 3.18E-02 |

Table 2. Experimental synthetic lethal pairs with p-values. Total of 20 hypothesis gene pairs were tested for synthetic lethality using SGA technology focusing on two pathways DNA topology change and regulation of protein biosynthesis. We confirmed total of 14 interactions with accuracy of 70%. Blue labeled pairs are confirmed pairs and red labeled pairs unconfirmed.

| Interaction ontology term | count |
| --- | --- |
| functional_relationship | 1333014 |
| physical_interaction | 185610 |
| non_covalent_binding | 185604 |
| complex | 155612 |
| interaction_pathway | 51307 |
| metabolic_interaction | 49894 |
| shared_pathway | 41013 |
| synthetic_interaction | 38439 |
| phenotypic_interaction | 20392 |
| synthetic_aggravation | 17166 |
| phenotypic_alleviation | 16023 |
| regulatory_interaction | 13194 |
| same_enzyme_class | 11937 |
| synthetic_lethal | 10374 |
| transcriptional_regulation | 8029 |
| synthetic_growth_defect | 7781 |
| posttranslational_regulation | 5049 |
| mediated_by_small_molecule | 4982 |
| covalent_modification | 4946 |
| synthetic_rescue | 4880 |
| synthetic_alleviation | 4880 |
| functional_group_transfer | 4805 |
| phosphate_transfer | 4740 |
| phosphorylation | 4701 |
| phenotypic_aggravation | 4484 |
| isoenzyme | 2560 |
| posttranscriptional_regulation | 129 |
| peptide_transfer | 107 |
| ubiquitin_transfer | 87 |
| Ubiquitination | 73 |

Table 3. Gold standard example count (only positive examples) for each interaction term.

| PubMedID | Publication year |
| --- | --- |
| 19966303 | 2009 |
| 19910927 | 2009 |
| 19860830 | 2009 |
| 19823668 | 2009 |
| 19706603 | 2009 |
| 19618914 | 2009 |
| 19581288 | 2009 |
| 19578373 | 2009 |
| 19457865 | 2009 |
| 19416974 | 2009 |
| 19398585 | 2009 |
| 19359360 | 2009 |
| 19358899 | 2009 |
| 19346402 | 2009 |
| 19303851 | 2009 |
| 19303850 | 2009 |
| 19270162 | 2009 |
| 19158380 | 2009 |
| 19153599 | 2009 |
| 19151091 | 2009 |
| 19106090 | 2009 |
| 19011240 | 2009 |
| 18939955 | 2009 |
| 18930846 | 2009 |

Table 4. Publication source list for posttranslational regulation analysis. The 24 publications combined experimentally confirmed 173 unique posttranslational gene pair interactions. We cross compared these new 173 pairs to our posttranslational regulation predictions on all gene pairs to result in an AUC score of 0.802.


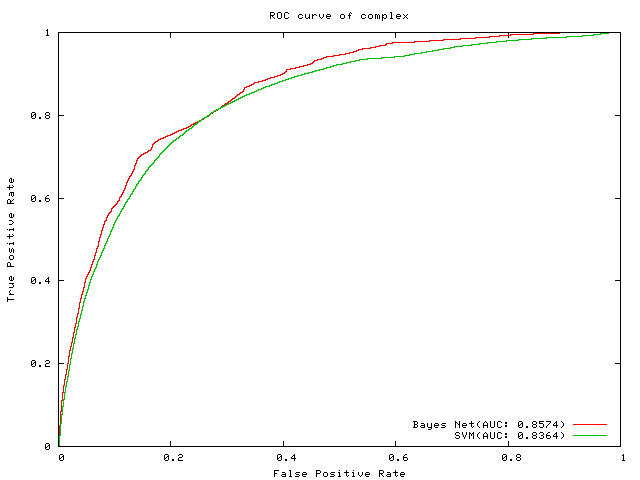


Figure 2. ROC curve for interaction term Complex consisting of the SVM output and result after Bayesian modification.


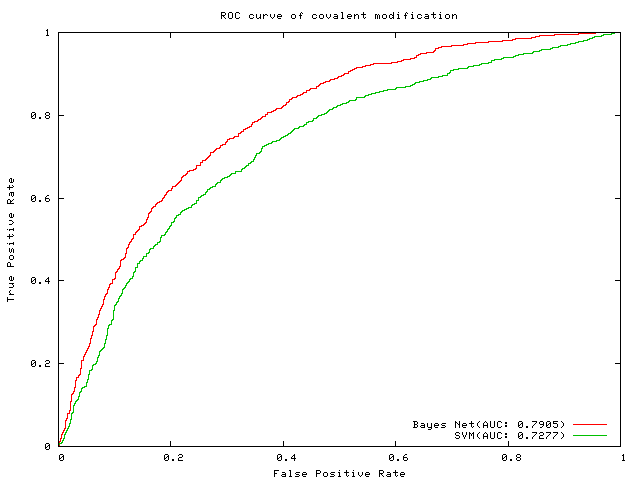


Figure 3. ROC curve for interaction term Covalent modification consisting of the SVM output and result after Bayesian modification.


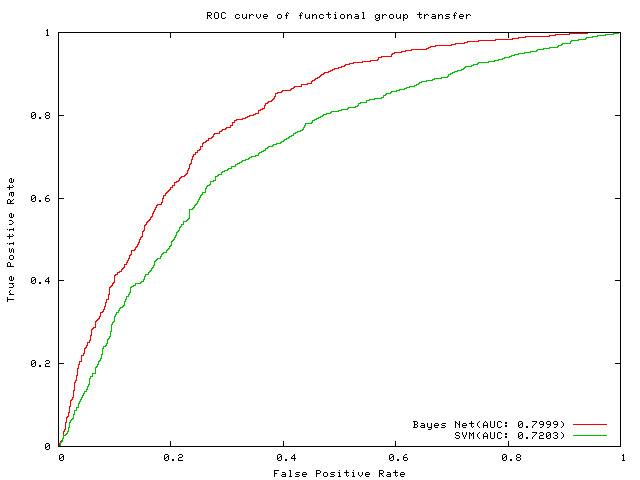


Figure 4. ROC curve for interaction term Functional group transfer consisting of the SVM output and result after Bayesian modification.


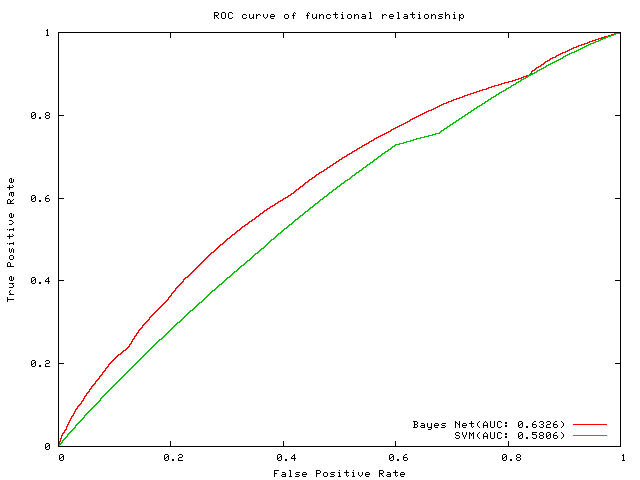


Figure 5. ROC curve for interaction term Functional relationship consisting of the SVM output and result after Bayesian modification.


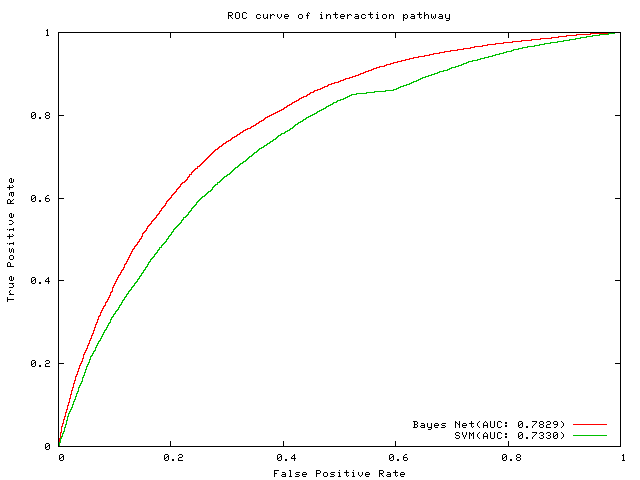


Figure 6. ROC curve for interaction term Interaction pathway consisting of the SVM output and result after Bayesian modification.


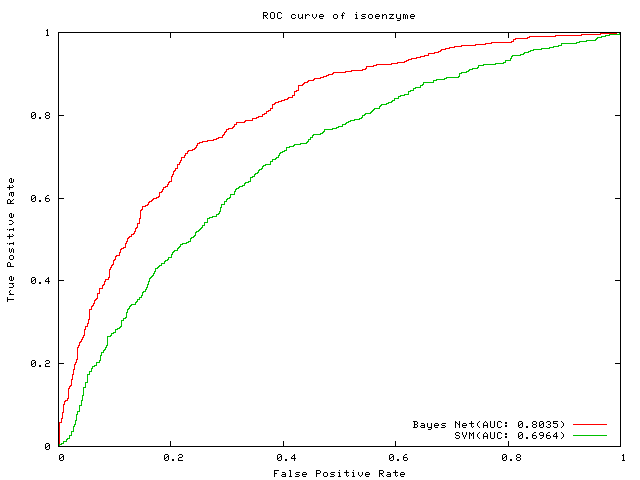


Figure 7. ROC curve for interaction term Isoenzyme consisting of the SVM output and result after Bayesian modification.


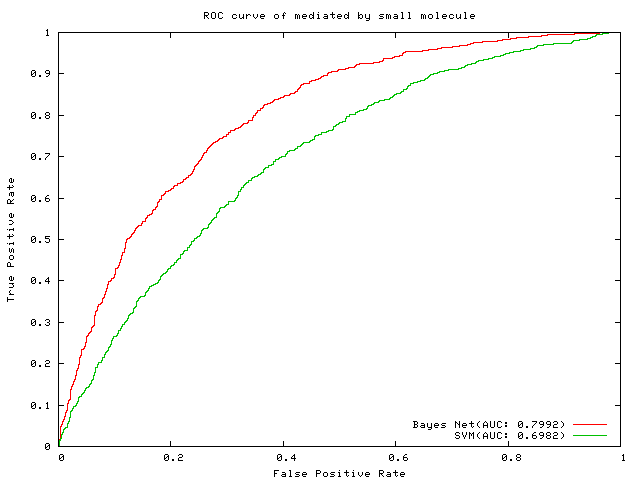


Figure 8. ROC curve for interaction term Small molecule consisting of the SVM output and result after Bayesian modification.


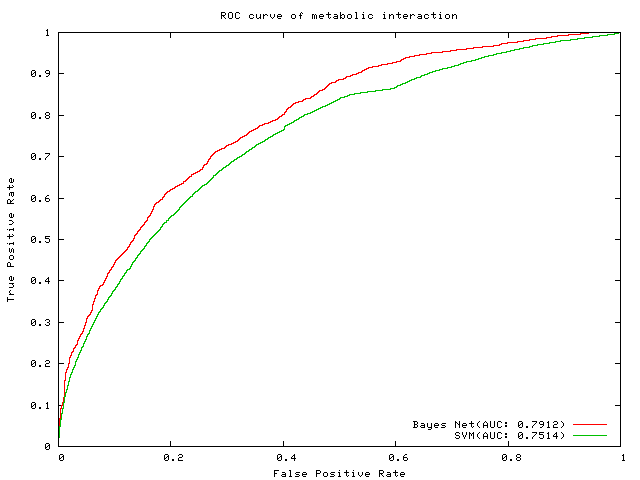


Figure 9. ROC curve for interaction term Metabolic interaction consisting of the SVM output and result after Bayesian modification.


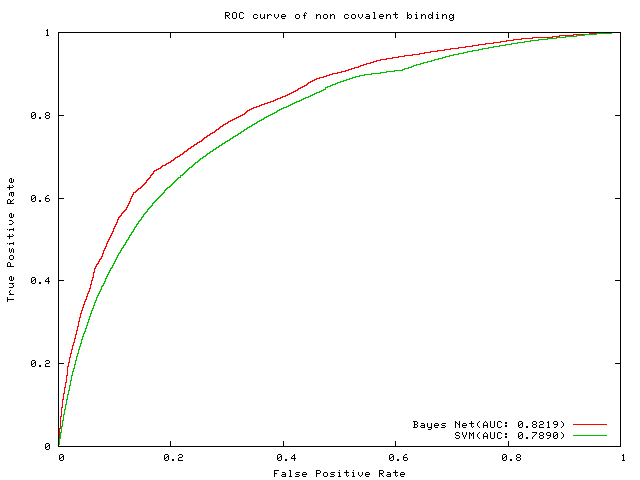


Figure 10. ROC curve for interaction term Non-covalent binding consisting of the SVM output and result after Bayesian modification.


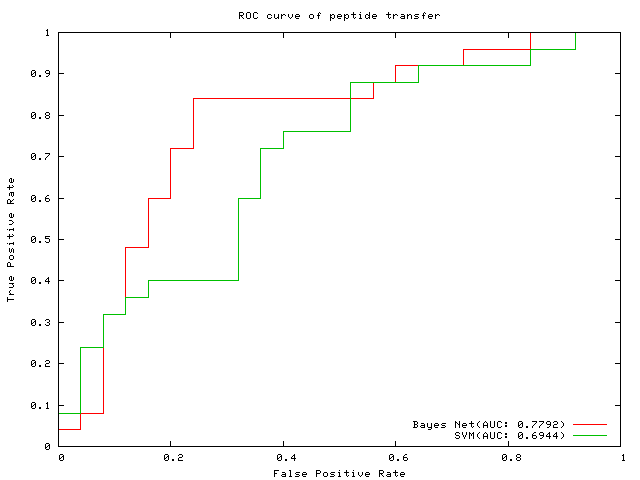


Figure 11. ROC curve for interaction term Peptide transfer consisting of the SVM output and result after Bayesian modification.


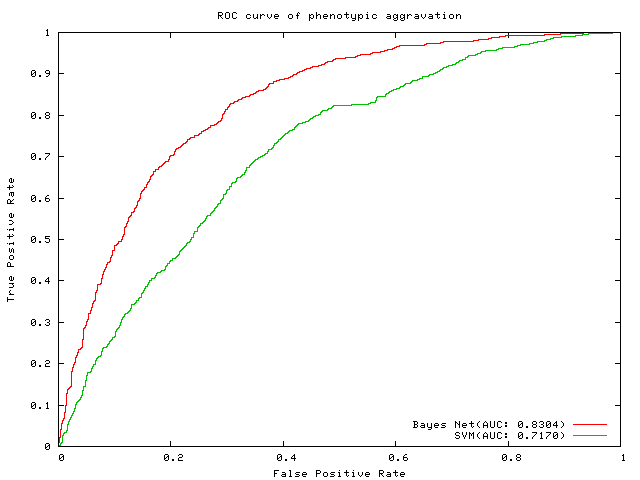


Figure 12. ROC curve for interaction term Phenotypic aggravation consisting of the SVM output and result after Bayesian modification.


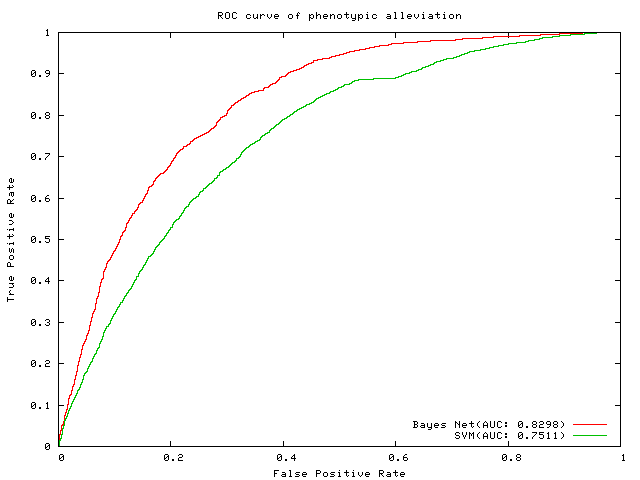


Figure 13. ROC curve for interaction term Phenotypic alleviation consisting of the SVM output and result after Bayesian modification.


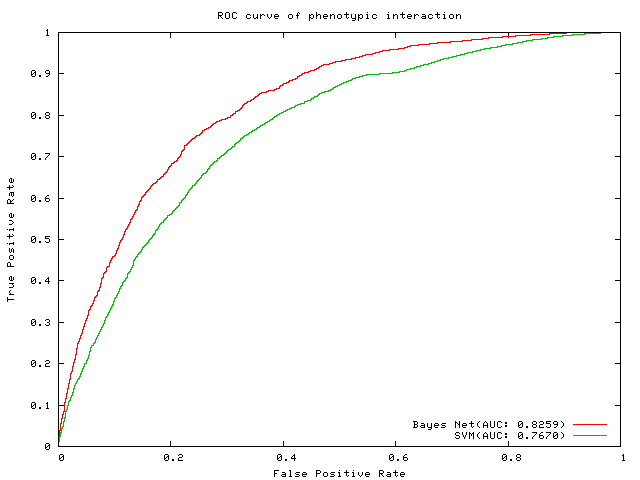


Figure 14. ROC curve for interaction term Phenotypic interaction consisting of the SVM output and result after Bayesian modification.


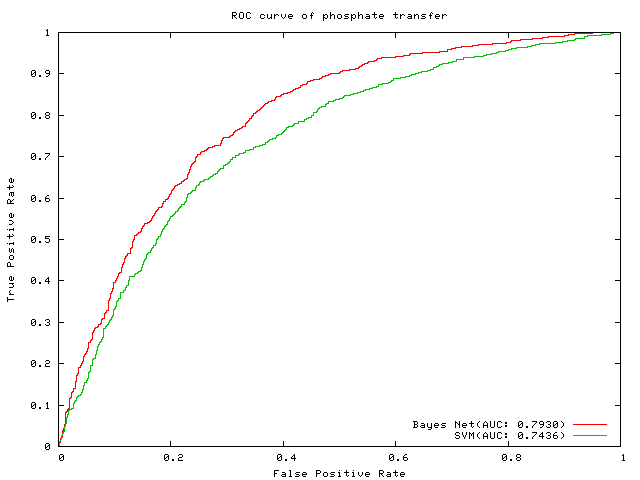


Figure 15. ROC curve for interaction term Phosphate transfer consisting of the SVM output and result after Bayesian modification.


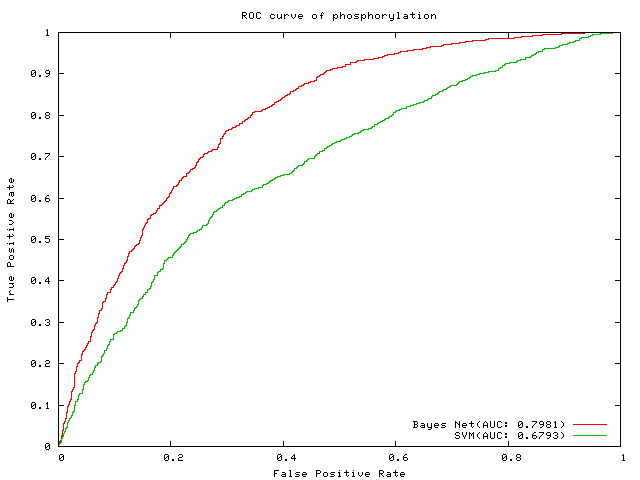


Figure 16. ROC curve for interaction term Phosphorylation consisting of the SVM output and result after Bayesian modification.


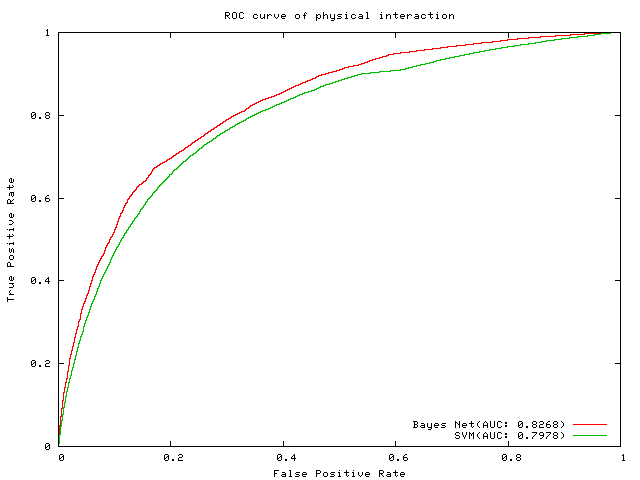


Figure 17. ROC curve for interaction term Physical interaction consisting of the SVM output and result after Bayesian modification.


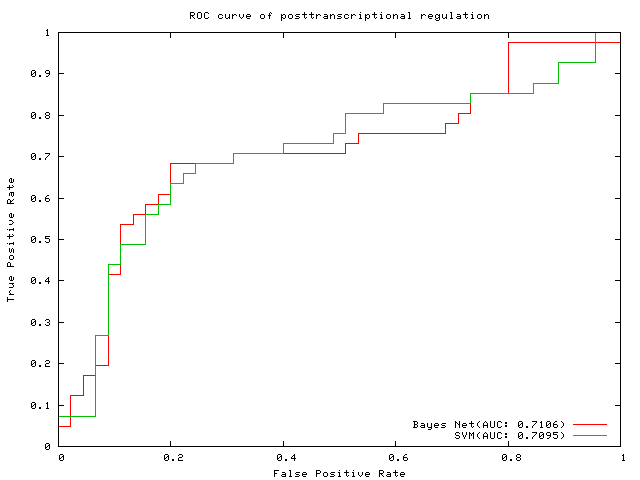


Figure 18. ROC curve for interaction term Posttranscriptional regulation consisting of the SVM output and result after Bayesian modification.


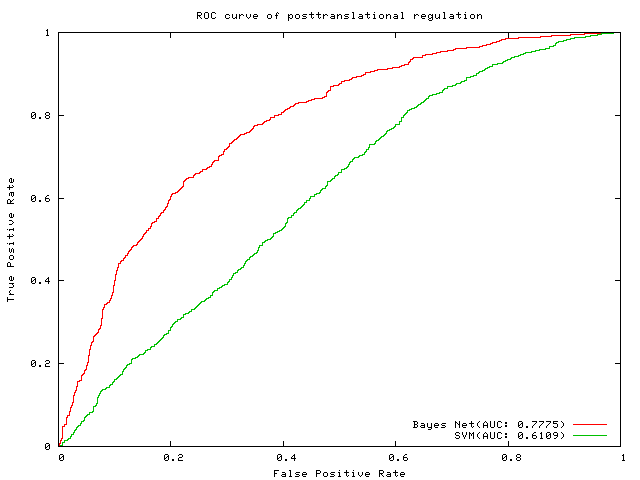


Figure 19. ROC curve for interaction term Posttranslational regulation consisting of the SVM output and result after Bayesian modification.


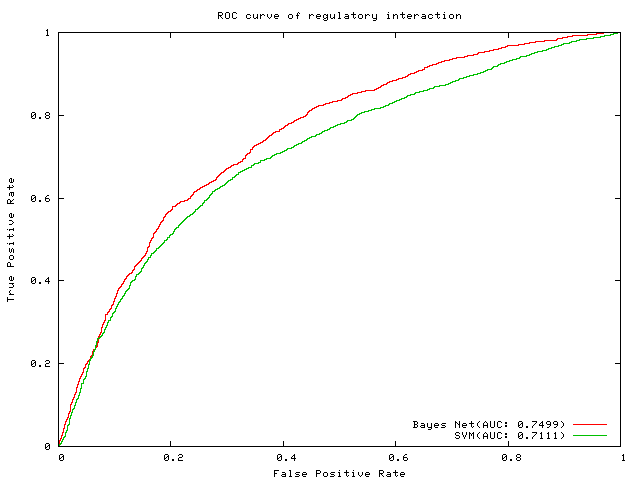


Figure 20. ROC curve for interaction term Regulatory interaction consisting of the SVM output and result after Bayesian modification.


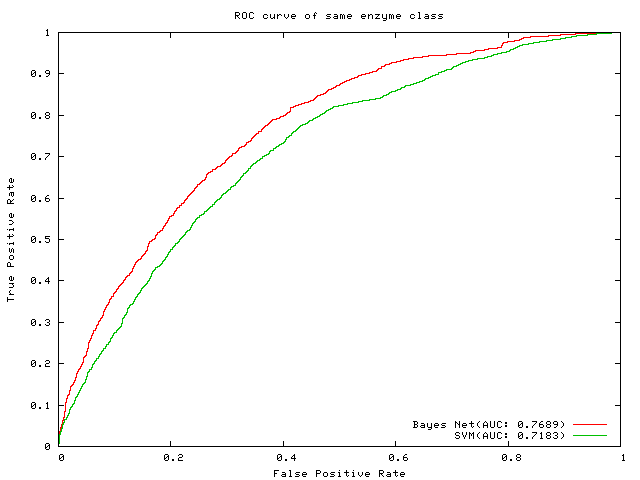


Figure 21. ROC curve for interaction term Same enzyme class consisting of the SVM output and result after Bayesian modification.


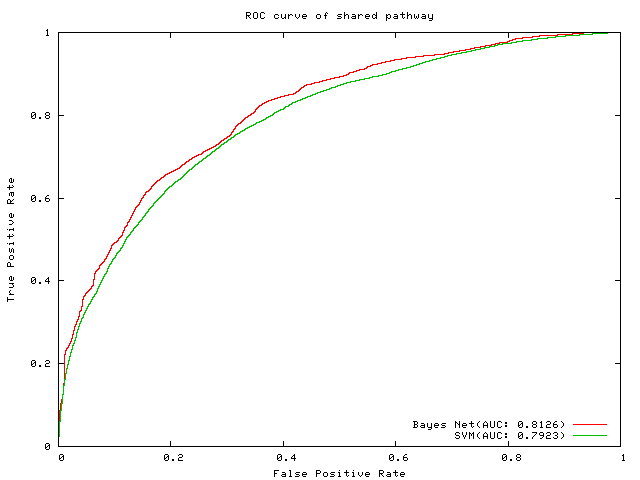


Figure 22. ROC curve for interaction term Shared pathway consisting of the SVM output and result after Bayesian modification.


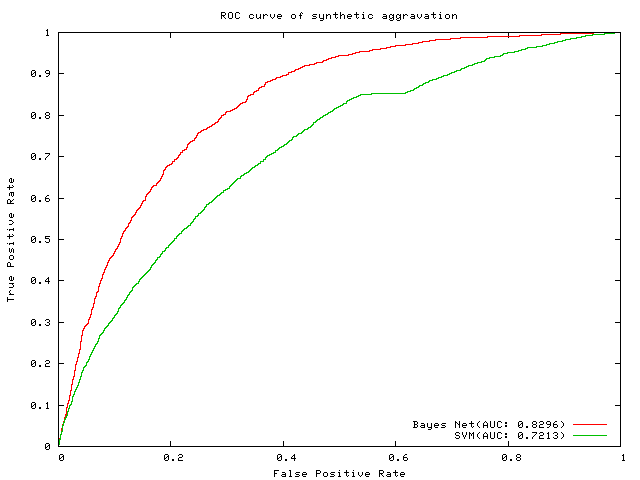


Figure 23. ROC curve for interaction term Synthetic aggravation consisting of the SVM output and result after Bayesian modification.


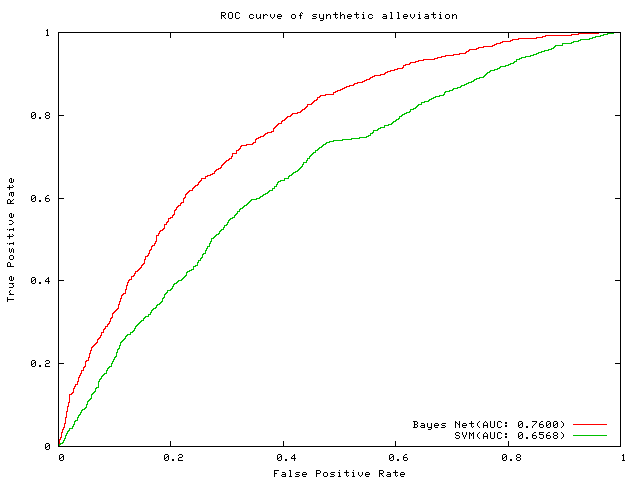


Figure 24. ROC curve for interaction term Synthetic alleviation consisting of the SVM output and result after Bayesian modification.


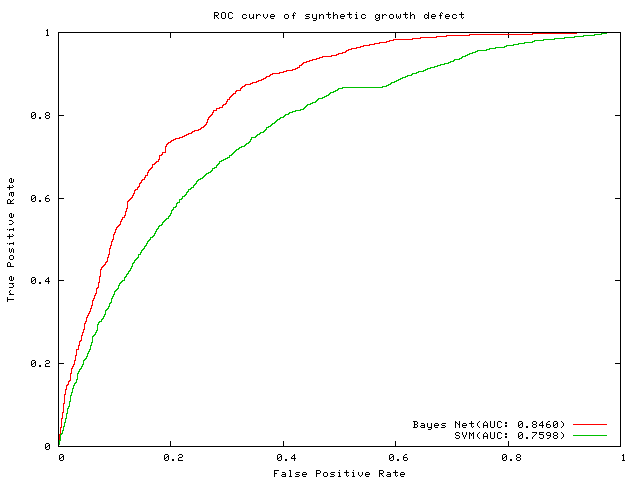


Figure 25. ROC curve for interaction term Synthetic growth defect consisting of the SVM output and result after Bayesian modification.


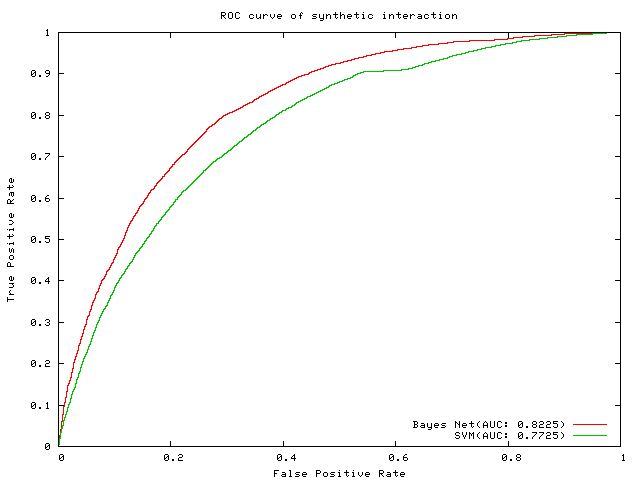


Figure 26. ROC curve for interaction term Synthetic interaction defect consisting of the SVM output and result after Bayesian modification.


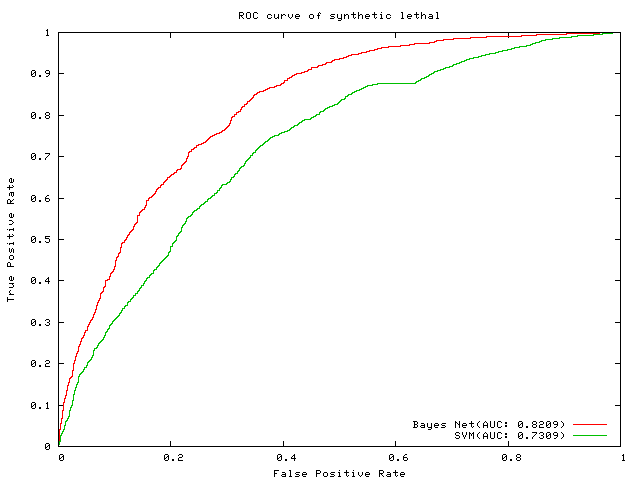


Figure 27. ROC curve for interaction term Synthetic lethal defect consisting of the SVM output and result after Bayesian modification.


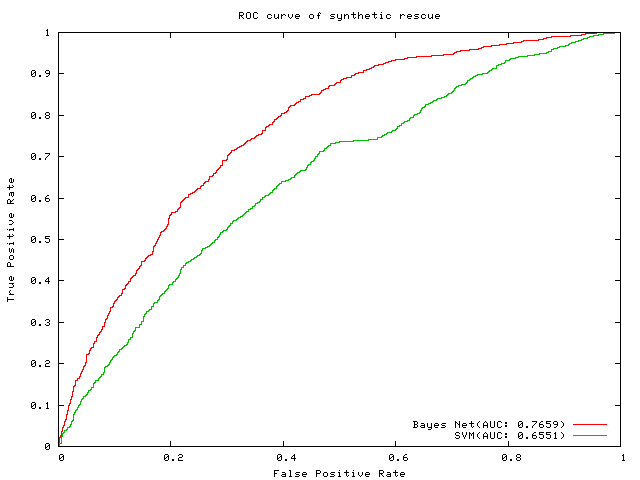


Figure 28. ROC curve for interaction term Synthetic rescue defect consisting of the SVM output and result after Bayesian modification.


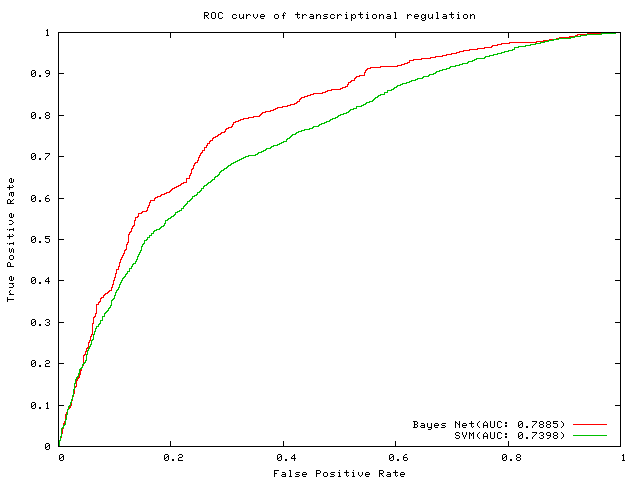


Figure 29. ROC curve for interaction term Transcriptional regulation defect consisting of the SVM output and result after Bayesian modification.


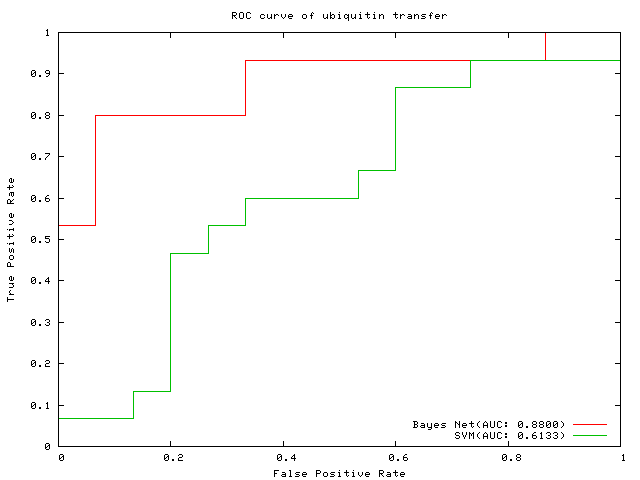


Figure 30. ROC curve for interaction term Ubiquitin transfer defect consisting of the SVM output and result after Bayesian modification.


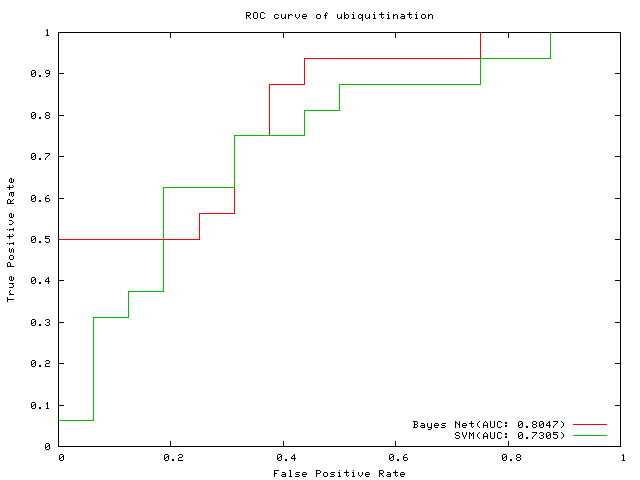


Figure 31. ROC curve for interaction term Ubiquitination defect consisting of the SVM output and result after Bayesian modification.

|  | Feed forward loop z-scores | original network frequency | average random network frequency |
| --- | --- | --- | --- |
| covalent_modification | 41.02 | 3.220% | 2.500% |
| functional_group_transfer | 41.496 | 3.226% | 2.475% |
| mediated_by_small_molecule | 43.695 | 3.279% | 2.508% |
| phosphate_transfer | 42.724 | 3.198% | 2.460% |
| phosphorylation | 43.798 | 3.203% | 2.458% |
| posttranscriptional_regulation | 16.132 | 3.549% | 3.125% |
| posttranslational_regulation | 18.031 | 3.154% | 2.723% |
| regulatory_interaction | 21.536 | 3.514% | 2.930% |
| transcriptional_regulation | 17.86 | 3.219% | 2.781% |
| peptide_transfer | -9.1303 | 2.519% | 2.687% |
| ubiquitin_transfer | -27.999 | 2.000% | 2.573% |
| ubiquitination | -12.645 | 2.187% | 2.432% |

Table 5. Feed forward loop motif enrichment z-scores for 12 directed interactomes. Across directed interactomes, excluding the ubiquitination interactomes, the feed forward loop motif showed significant enrichment compared to 500 random networks that had conserved edge degree for each gene.


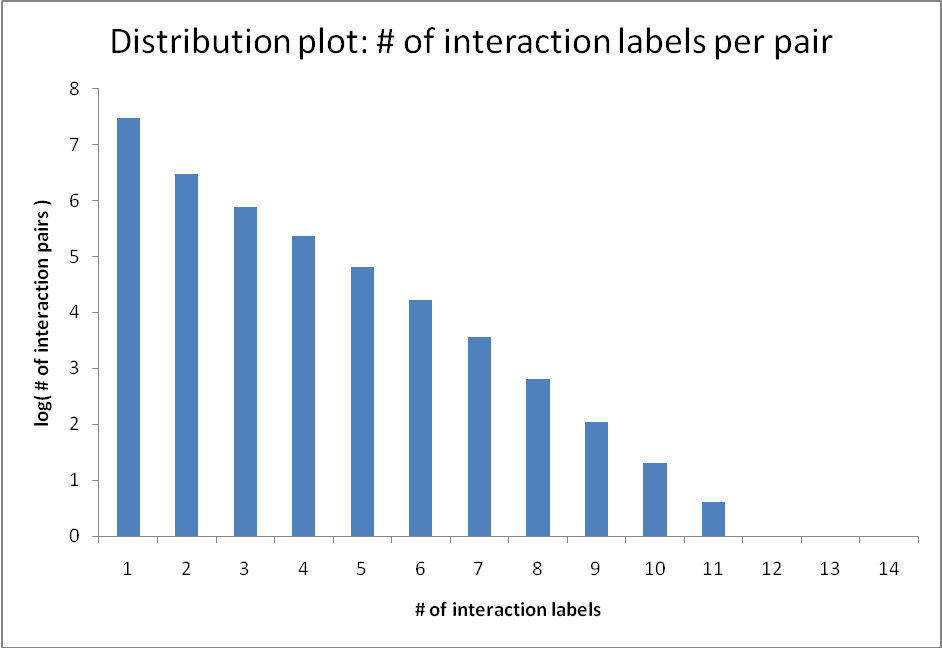


Figure 32. The distribution of # protein pairs per # interaction leaf term labels. We plotted the number of protein pairs that were labeled with various number of leaf node interaction labels. Roughly there is an exponential decrease in protein pairs as you increase the number of interaction labels with an average of 0.165 interaction labels per protein pair.
