# Supplementary figures and images for: Simultaneous Genome-Wide Inference of Physical, Genetic, Regulatory, and Functional Pathway Components
Source: PLoS Comput Biol. 2010 Nov 24;6(11):e1001009. doi: 10.1371/journal.pcbi.1001009 (PMC2991250; doi:10.1371/journal.pcbi.1001009)

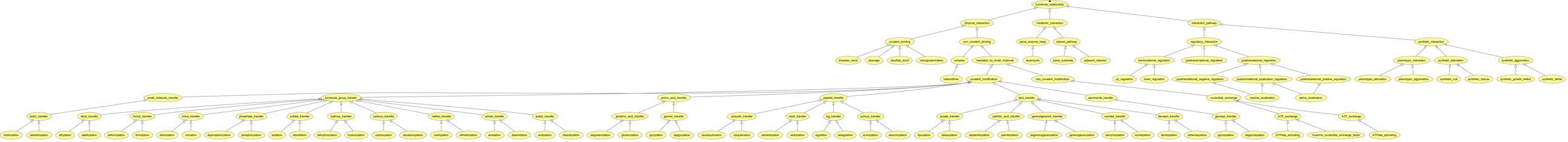

Supplement: Text S3 — Interaction ontology files - includes OWL ontology format file and visual ontology PDF file (0.03 MB ZIP) [file pcbi.1001009.s003.zip › interaction_ontology.pdf]
